# Graphene: Preparation, tailoring, and modification

**DOI:** 10.1002/EXP.20210233

**Published:** 2023-01-19

**Authors:** Mingyao Li, Bing Yin, Chunyan Gao, Jie Guo, Cong Zhao, Chuancheng Jia, Xuefeng Guo

**Affiliations:** ^1^ Beijing National Laboratory for Molecular Sciences, National Biomedical Imaging Center, College of Chemistry and Molecular Engineering Peking University Beijing China; ^2^ Center of Single‐Molecule Sciences, Institute of Modern Optics, Tianjin Key Laboratory of Micro‐scale Optical Information Science and Technology, Frontiers Science Center for New Organic Matter, College of Electronic Information and Optical Engineering Nankai University Tianjin China

**Keywords:** device, etching, exfoliation, graphene, modification

## Abstract

Graphene is a 2D material with fruitful electrical properties, which can be efficiently prepared, tailored, and modified for a variety of applications, particularly in the field of optoelectronic devices thanks to its planar hexagonal lattice structure. To date, graphene has been prepared using a variety of bottom–up growth and top–down exfoliation techniques. To prepare high‐quality graphene with high yield, a variety of physical exfoliation methods, such as mechanical exfoliation, anode bonding exfoliation, and metal‐assisted exfoliation, have been developed. To adjust the properties of graphene, different tailoring processes have been emerged to precisely pattern graphene, such as gas etching and electron beam lithography. Due to the differences in reactivity and thermal stability of different regions, anisotropic tailoring of graphene can be achieved by using gases as the etchant. To meet practical requirements, further chemical functionalization at the edge and basal plane of graphene has been extensively utilized to modify its properties. The integration and application of graphene devices is facilitated by the combination of graphene preparation, tailoring, and modification. This review focuses on several important strategies for graphene preparation, tailoring, and modification that have recently been developed, providing a foundation for its potential applications.

## Graphical abstract

Graphene is a key material for a new generation of electronic devices with unique electrical properties. In this review, current advances in the preparation, tailoring, and modification of graphene to obtain high‐quality graphene with precisely controlled patterning and specific functional groups are summarized.

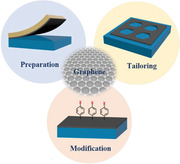

## 1 INTRODUCTION

Graphene, which consists of 2D sp^2^‐hybridized carbon, has been intensively studied nowadays. Physicists and chemists have been drawn to graphene due to its unique properties, such as high mechanical strength,^[^ ^1^ ^]^ high electrical conductivity,^[^ ^2^, ^3^, ^4^, ^5^ ^]^ and large surface area.^[^ ^6^, ^7^, ^8^ ^]^ These excellent properties support the use of graphene for various electrical and optical applications. Effective approaches to graphene preparation, tailoring, and modification are required to realize these applications.

Graphene can be produced by both physical and chemical methods. The preparation methods of graphene can be classified into two types: bottom‐up and top‐down methods.^[^ ^9^, ^10^ ^]^ Bottom‐up methods depend upon chemical reactions of precursors and produce large‐area graphene with controlled single layer, which is suitable for building large‐area electronic devices.^[^ ^11^, ^12^, ^13^ ^]^ Top‐down methods, consisting of mechanical exfoliation,^[^ ^14^, ^15^, ^16^ ^]^ liquid‐phase exfoliation,^[^ ^17^, ^18^, ^19^, ^20^ ^]^ and so on, are proposed based on the unique structure of graphite, where carbon atoms in the sp^2^ hybrid manner form stable covalent bonds and the adjacent layers are combined through weak van der Waals attraction.^[^ ^21^, ^22^, ^23^ ^]^ Graphene flakes can be peeled from bulk graphite once the van der Waals attraction weakens. Top‐down methods are simpler and more convenient than bottom‐up methods because they do not require complex condition control or expensive equipment. More importantly, top‐down methods are suitable for building high‐performance devices to study the intrinsic characteristics of graphene.

It is necessary to tailor graphene into various shapes in order to process and integrate it into electronic devices. For various functional devices, graphene etching is the primary method for patterning desired shapes and controlling layer numbers. Traditional etching techniques in the semiconductor industry, such as photolithography,^[^ ^24^, ^25^ ^]^ electron beam lithography (EBL),^[^ ^26^, ^27^ ^]^ plasma etching,^[^ ^28^, ^29^, ^30^ ^]^ and so on, have been making great advancement and the precision has been improved to the nanoscale, leading to the rapid progress of microelectronic integrated circuits. However, conventional etching methods cannot achieve atomic‐level precision, which plays a crucial role in the fabrication of devices with uniform edge‐configuration, especially in spintronic devices.^[^ ^31^, ^32^ ^]^ Gas etching has been discovered to have the feature of anisotropic etching, which can achieve atomic‐level precision, and may provide opportunities for precise processing.

Modification is necessary to endow graphene with various properties and functionalities, including noncovalent and covalent modifications to further realize the functional applications. The noncovalent modification uses π–π interactions to modify graphene surface, while covalent modification uses chemical bonds to decorate both the plane and edge of graphene, becoming a more stable and reliable method.^[^ ^33^, ^34^, ^35^, ^36^ ^]^ Many organic reactions are applicable to graphene due to the richness of carbon chemistry. Electrophilic substitution reactions,^[^ ^37^, ^38^ ^]^ free radical reactions,^[^ ^39^, ^40^ ^]^ cycloaddition reactions,^[^ ^41^, ^42^ ^]^ and coupling reactions^[^ ^43^, ^44^ ^]^ are four main approaches, which are classified according to the types of chemical reactions. In addition, mechanochemical methods mainly make use of ball milling^[^ ^45^ ^]^ to break the graphic C─C framework and introduce the target groups, which is an effective and controllable method. The modifications are divided into two types based on the reaction sites: edge and surface modifications. Ball‐milling methods^[^ ^46^ ^]^ and Friedel–Crafts acylation^[^ ^47^ ^]^ are the most common methods for edge modification. Edge modification has been considered as a reliable way to tune its electronic and mechanical properties without damaging its conjugated π‐electron system.^[^ ^48^ ^]^ Surface modification of graphene can more effectively adjust the band gap^[^ ^49^ ^]^ and introduce the band level, resulting in more applications in the nanoelectronics fields. In comparison to pristine graphene, the modified graphene has outstanding characteristics and can be used for a variety of applications.

**FIGURE 1:**
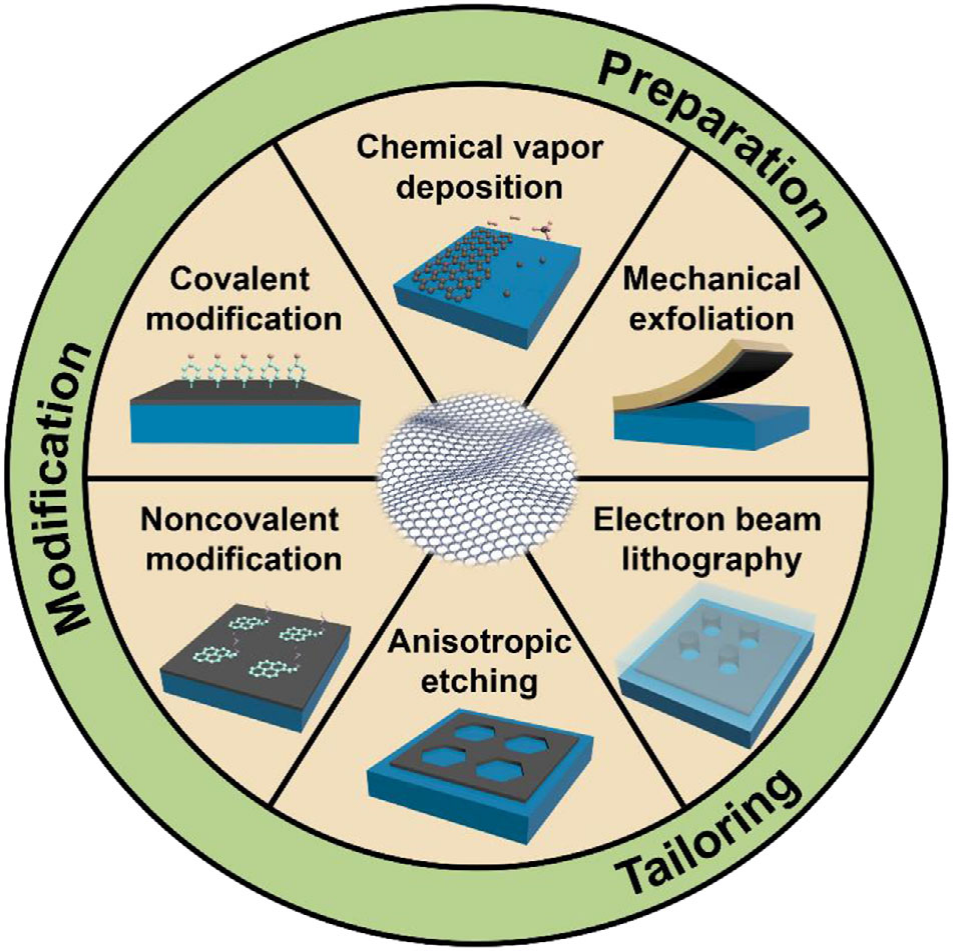
Schematic illustration of main techniques for graphene preparation, tailoring, and modification

In this review, we pay attention to the recent significant development of graphene preparation, tailoring, and modification, supplemented by device fabrication (Figure 1). First, methods of graphene preparation are illustrated, which is the foundation for high‐quality graphene to fabricate electronic devices. Then, graphene tailoring, particularly anisotropic etching, is then used to control the morphology and edge configuration of graphene, making it easier to process and integrate. Finally, recent works on graphene modification are presented, providing a promising way to change the inertness of the edge and basal‐plane for advanced properties. These three steps are important for the physical and chemical processing of graphene, and they form the foundation for graphene's use in devices and other applications.

## 2 PREPARATION OF GRAPHENE

The exploration of the property and further application of graphene depends on the development of preparation methods, which can be roughly divided into bottom‐up and top‐down methods, represented by chemical vapor deposition (CVD) and mechanical exfoliation, respectively. High‐density defects and wrinkles in graphene produced by CVD reduce its performance in electronic devices. The mechanical exfoliation method can directly exfoliate bulk graphite layer by layer to produce graphene with high‐quality, which is essential for the discovery of new phenomena and the development of high‐performance devices. Many efforts, such as optimizing deposition conditions, have been made to produce high‐quality graphene via CVD, but there are still challenges in achieving the same high quality as mechanically exfoliated graphene. However, most exfoliation methods produce small, low‐yield graphene flakes, limiting device fabrication efficiency and application possibilities. Mechanical exfoliation can be tweaked to address the aforementioned drawbacks. In the following part, we mainly discuss the improvement of mechanical exfoliation (Table 1) after a brief introduction of CVD (Table 2).

**TABLE 1:** Comparison between different exfoliation methods

Exfoliation methods	Size	Quality	Thickness	Complexity	Ref.
Scotch tape exfoliation	∼10 μm	High	Random	Easy	[14]
Modified mechanical exfoliation	∼500 μm	High	Monolayer	Moderate	[78]
Extrinsic corrugation‐assisted exfoliation	∼100 μm	Medium	Monolayer	Moderate	[79]
Anode bonding exfoliation	∼300 μm	Low	Monolayer	Difficult	[80]
Metal‐assisted exfoliation	∼1 mm	Medium	Controllable	Difficult	[84, 85]

**TABLE 2:** Comparison between different CVD methods

Substrate	Precursor	Temperature	Grain size	Number of layers	Ref.
Cu	CH_4_	1000°C	> 100μm	Monolayer	[51]
Ni	CH_4_	1000°C	> 400μm	Monolayer	[61]
Cu/Ni alloy	C_2_H_4_ CH_4_	727−757°C 1075°C	Wafer‐scale > 150 μm	Monolayer Bilayer/Trilayer	[63] [62]
SiO_2_	CH_4_	1185°C	∼1μm	Monolayer	[73]
Sapphire	CH_4_	1050°C	Wafer‐scale	Monolayer	[74]

### 2.1 Chemical vapor deposition

CVD has been widely applied to fabricate large‐area graphene films.^[^ ^50^, ^51^, ^52^, ^53^ ^]^ Under the catalysis of Cu, a typical catalyst for graphene growth, carbon species decomposed from hydrocarbons (usually CH_4_) nucleate on Cu, and the nuclei further grow into large domains until the surface of Cu foil is completely covered.^[^ ^51^ ^]^ Large‐area monolayer graphene can be made because of the poor solubility of carbon in Cu.^[^ ^54^ ^]^ Graphene films are often polycrystalline because of the misalignment of the domains.^[^ ^55^, ^56^, ^57^, ^58^ ^]^ Moreover, wrinkles arising from the mismatch of thermal expansion coefficient between graphene and Cu are commonly observed.^[^ ^59^, ^60^ ^]^ Different from the self‐limiting mechanism on Cu foils, the growth of graphene on Ni is mainly based on the precipitation and segregation of dissolved C atoms because of its better solubility of C and higher catalytical reactivity, which often results in inhomogeneous and multilayer graphene.^[^ ^61^ ^]^ In combination with the advantages of Cu and Ni, the Cu/Ni alloy has been intensively studied for CVD growth of graphene due to its good and controllable solubility of C. As the Ni content in the alloy increases, single‐crystal bilayer, trilayer, and even multilayer graphene can be prepared.^[^ ^62^ ^]^ In addition, large‐area and adlayer‐free monolayer graphene can also be achieved on Cu–Ni (111) alloy foils.^[^ ^63^ ^]^ For the preparation of high‐quality graphene, many factors including substrates, carbon sources, and growth conditions must be carefully considered and controlled.^[^ ^64^, ^65^ ^]^ CVD is also widely used to produce large‐scale graphene films to meet the requirements of graphene industrialization for display and sensing applications. Automated transfer processes, such as roll‐to‐roll,^[^ ^66^ ^]^ are applied to improve transfer efficiency and avoid quality degradation, which is superior to wet etch transfer used in laboratories.

Graphene grown on Cu foils often needs to be transferred to a target substrate for the fabrication of electronic devices. Wet transfer is the most common transfer method.^[^ ^66^, ^67^, ^68^ ^]^ Usually, poly(methyl methacrylate) (PMMA) can be served to support graphene as a carrier, and copper foil is etched away by chemical etchant (FeCl_3_).^[^ ^67^ ^]^ The PMMA/graphene stack is picked up by a target substrate after floating on the surface of the etchant solution. After PMMA is dissolved with acetone, the graphene remains on the substrate. However, the contacts between PMMA and graphene surfaces are evitable, and there are difficulties in the complete removal of PMMA.^[^ ^69^, ^70^, ^71^ ^]^ For instance, acetone cannot entirely dissolve PMMA, and a thin layer of residues may cause degradation of the carrier mobility in graphene.^[^ ^72^ ^]^

In order to prevent contaminations and wrinkles introduced during wet transfer, the growth of graphene on insulators and semiconductors, such as SiO_2_ and sapphire (Al_2_O_3_), is vital for achieving high‐performance graphene‐based devices. Common approaches of direct growth on SiO_2_/Si require relatively high temperature, typically over 1100°C, resulting in low‐quality graphene due to the instability of SiO_2_.^[^ ^73^ ^]^ With the assistance of enhanced plasma, the temperature of graphene growth on SiO_2_/Si can be effectively lowered. Recently, a sapphire‐based multi‐cycle plasma etching‐assisted CVD method successfully led to the growth of wafer‐scale single‐crystal monolayer graphene, which produces graphene via a sacrificial layer of Cu rather than growing directly on sapphire.^[^ ^74^ ^]^ After a long annealing process, the copper grains are transformed into a regular orientation through which C atoms diffuse into the interface between Cu (111) and Al_2_O_3_ (0001). SiC is also a promising insulated substrate for obtaining graphene. At high temperatures above 1600°C, Si atoms on the SiC surface are sublimated, while C atoms are remained to grow into high‐quality graphene.^[^ ^75^ ^]^

### 2.2 Mechanical exfoliation

Mechanical exfoliation, in contrast to stronger in‐plane covalent bonding, relies on the relatively weak interlayer van der Waals forces, resulting in exfoliated flakes with a wide range of thickness and lateral dimensions. Though mechanical exfoliation can avoid defects caused by chemical synthesis and contamination introduced by wet transfer, the thickness, sizes, shapes, and locations of the obtained graphene are usually random, limiting the large‐scale device fabrication. Because of the disadvantages mentioned above, mechanical exfoliation is still a long way from mass production and commercialization, but it remains the best way to obtain high‐quality graphene for fundamental studies.

#### 2.2.1 Scotch tape exfoliation

**FIGURE 2:**
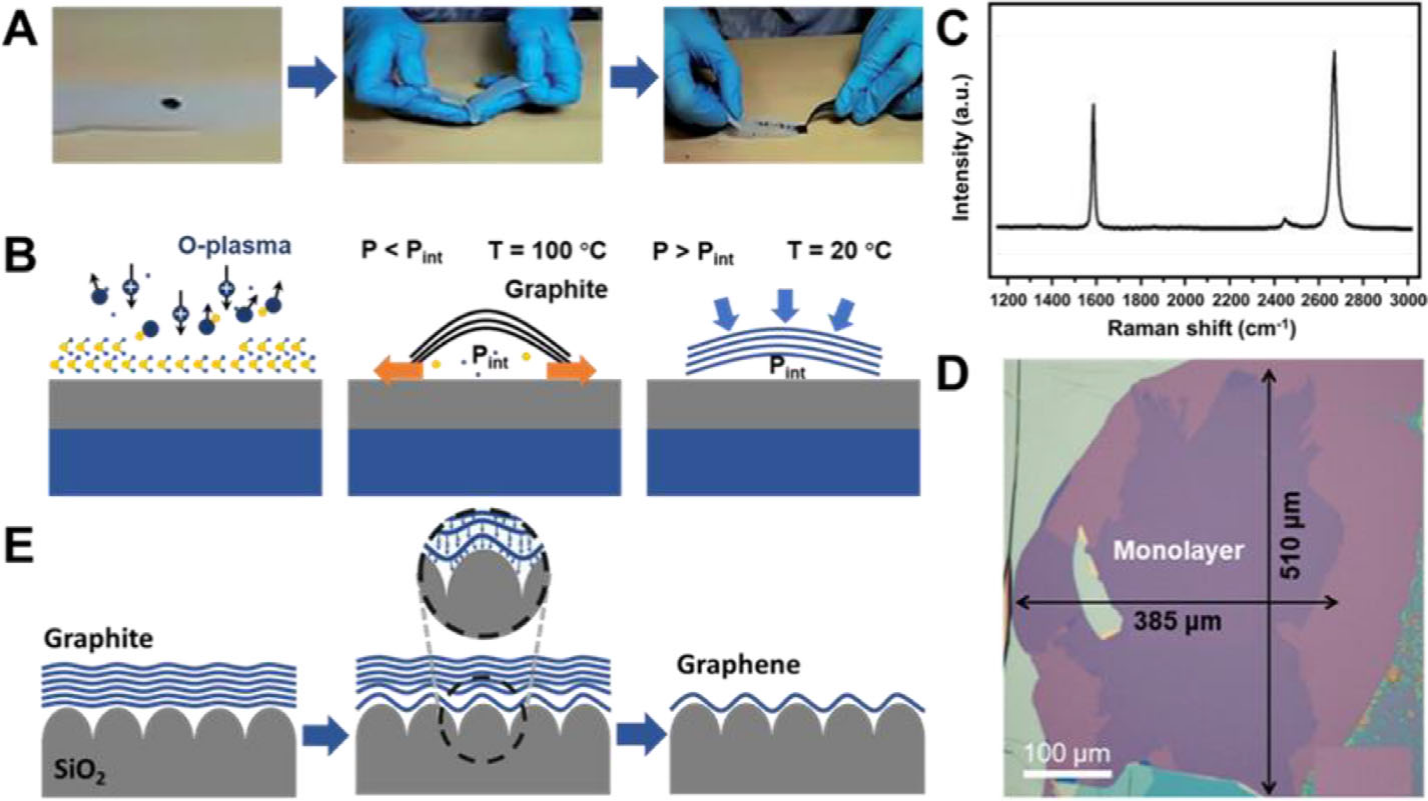
(A) Schematic illustration of traditional Scotch tape exfoliation process. Reproduced with permission.^[^ ^76^ ^]^ Copyright 2015, The Royal Society of Chemistry. (B) Key procedures of modified exfoliation method, including O_2_ plasma cleaning and mild annealing. (C,D) Raman spectroscopy and optical micrograph of a monolayer graphene flake prepared by modified exfoliation method. Reproduced with permission.^[^ ^78^ ^]^ Copyright 2015, American Chemical Society. (E) Process of extrinsic corrugation‐assisted exfoliation method. Reproduced with permission.^[^ ^79^ ^]^ Copyright 2010, Wiley‐VCH

In 2004, Geim and Novoselov et al. first isolated graphene with Scotch tape from highly oriented pyrolytic graphite (HOPG).^[^ ^14^ ^]^ This exfoliation process is illustrated in Figure 2A.^[^ ^76^ ^]^ First, a piece of graphite is placed on the Scotch tape. After folding the tape in half several times to fully exfoliate the bulk crystal, it is pressed with the proper force on a Si wafer. Finally, single‐layer graphene is obtained by peeling the tape away from the wafer at a slow speed. This method is widely used in laboratories thanks to its advantages of producing high‐quality graphene, and it has also emerged as a promising method for obtaining other 2D materials, especially transition metal dichalcogenides (TMDs).^[^ ^77^ ^]^

The mechanical exfoliation depends on two kinds of forces, lateral force and normal force. The lateral force is derived from graphite's ability to self‐lubricate, which aids the movement between adjacent layers. Few‐layer graphene can be obtained by overcoming the van der Waals force between adjacent layers via the normal force. During the process of exfoliation, uneven interactions can cause the breakdown of in‐plane covalent bonds, producing fragmented small‐area graphene.

The standard exfoliation method involves repeatedly folding Scotch tape to obtain graphene with random number of layers; thus, graphene quality and exfoliation yield are affected by a number of factors, including the substrate, the tape, the experimenters’ operation, temperature, and humidity. Among these factors, the tape and substrate play a vital role in improving peeling efficiency. The modification of substrate and tape is mostly considered to reduce the influence of uncontrollable factors (e.g., the operation of the experimenters, temperature, and humidity) and improve the exfoliation yield.

#### 2.2.2 Modified mechanical exfoliation

In order to homogenize and enhance the adhesion force between the substrate and graphene, a modified mechanical exfoliation method is developed.^[^ ^78^ ^]^ The ambient adsorbates are removed via oxygen plasma, and followed by heat treatment to ensure uniform contact with the interface. The oxygen plasma cleaning and mild annealing are considered key steps in this method (Figure 2B). Organic adsorbates are removed by oxygen plasma, and annealing process creates a pressure difference to remove the gas in the gap, resulting in improved van der Waals interaction at the interface. Characterized by Raman spectroscopy, the monolayer graphene is confirmed to be of high‐quality (Figure 2C). These two simple procedures can increase the yield of graphene flakes 50 times higher than other established methods (Figure 2D).

#### 2.2.3 Extrinsic corrugation‐assisted exfoliation

At higher temperature, the heat treatment changes the morphology of the bottom layer graphene, which helps to release gas between the graphene sheet and substrate. An extrinsic corrugated‐assisted mechanical exfoliation method (ECAME) is reported.^[^ ^79^ ^]^ The thick graphite is placed on a SiO_2_/Si substrate and annealed for 2 h at 350°C to obtain large monolayer graphene. The process is simple, and the peeling efficiency can reach more than 60%. The successful exfoliation of monolayer graphene has been explained using a model (Figure 2E). The shape of bottom‐layer graphene is influenced by the long‐corrugation of Si wafer with a SiO_2_ layer. Once the bottom‐layer graphene is adapted to the shape of the corrugated surface, the contact between the underlayer sheet and substrate will be significantly enhanced, weakening the van der Waals forces with upper graphene layers. Thus, large monolayer graphene can be obtained after top graphite flakes are removed by an adhesive tape. With a patterned HOPG stamp pressed on the corrugated substrate and annealed at high temperature, the ECAME approach can be used to obtain patterned graphene, promoting the fabrication of graphene functional devices.

#### 2.2.4 Anode bonding exfoliation

The basic idea of anode bonding is to enhance the interaction between the bottom layer of graphite and the substrate via forming a strong electrical field.^[^ ^80^ ^]^ Under a kilovolt voltage, Na_2_O in the glass substrate decomposes into O^2−^ and Na^+^ ions at high temperatures. Na^+^ ions migrate across the substrate under the influence of an electric field due to their relatively high mobility, while O^2−^ ions are static, making the surface negatively charged. Thus, a strong electrical field is created, allowing for close atomic contact between graphite and substrate. The graphite is then cleaved away with an adhesive tape, leaving a few layers of graphene adhered to the substrate. The graphene produced can be hundreds of microns in size. However, this method requires high voltage (1.2−1.7 kV) and temperature (200−400°C), which is unavailable in most laboratories. The strong electrostatic effect makes it hard to transfer to other substrates, which may limit its further applications.

#### 2.2.5 Metal‐assisted exfoliation

It is difficult to produce large‐area single‐layer graphene with traditional exfoliation method due to uneven interactions between graphite, tape, and substrate. Recently, a noncovalent interaction (∼0.5 eV per unit cell) between some metals and 2D materials,^[^ ^81^, ^82^ ^]^ named covalent‐like quasi‐bonding, is considered to meet the requirements for mechanical exfoliation, which provides uniform and strong interaction with 2D materials.^[^ ^83^ ^]^ Only a few metals can help with graphene exfoliation due to differences in binding energies between metals and graphene. Ni can be used to peel graphene off due to its high binding energy.^[^ ^84^, ^85^ ^]^ While Au, widely used to peel off TMDs, is less effective for graphene exfoliation due to the low binding energy.^[^ ^86^, ^87^, ^88^, ^89^ ^]^ Metal‐assisted exfoliation becomes an ideal way to transfer materials tightly bound to the growth substrate, such as graphene grown on the silicon carbide (SiC) surface, due to the strong interactions between metal and the 2D materials.

**FIGURE 3:**
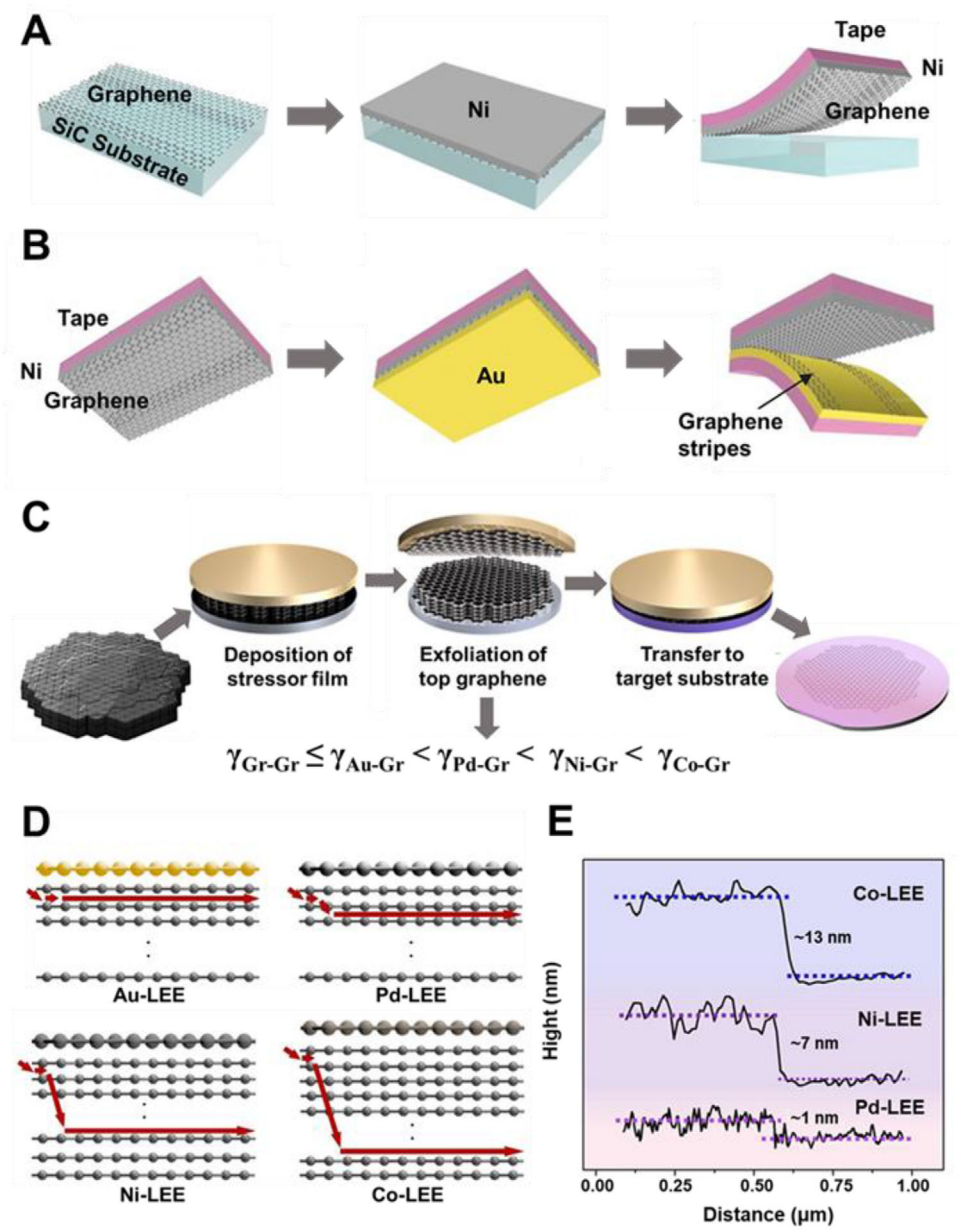
(A) Schematic of metal‐assisted exfoliation of graphene directly from the SiC surface with an adhesive‐strained Ni layer. (B) Schematic of the method for removing double‐layer stripes from the exfoliated graphene with a second adhesive‐strained Au layer. Reproduced with permission.^[^ ^84^ ^]^ Copyright 2013, American Association for the Advancement of Science. (C) Schematic of the layer‐engineered large‐area graphene exfoliation technique. (D) Schematic of the spalling path depending on different metal films. (E) Height profile of the graphene edges prepared by the layer‐engineered exfoliation method with different metals. Reproduced with permission.^[^ ^85^ ^]^ Copyright 2020, American Association for the Advancement of Science

Single‐oriented graphene can be obtained with the decomposition of SiC (0001) surface but is mostly one or two layers.^[^ ^90^, ^91^ ^]^ Due to the strong bonding of graphene to the SiC surface, transferring graphene from SiC is still challenging. A method for separating graphene from SiC has been proposed (Figure 3A).^[^ ^84^ ^]^ To peel graphene off of SiC, Ni film and thermal tape are used as the handling layer. After removing the thermal tape by heating and etching the Ni film with a mild etchant solution (FeCl_3_), the graphene layer is transferred to a SiO_2_/Si substrate. The exfoliation efficiency depends on the binding energy (γ_Gr‐Metal_) and the stress of the metal film. Under the same deposition conditions, Ni has the highest stress and the largest binding energy (γ_Gr‐Ni_) among other metals (γ_Gr‐Au_ < γ_Gr‐Cu_ < γ_Gr‐Pd_ < γ_Gr‐SiC_ < γ_Gr‐Ni_), which exceeds γ_Gr‐SiC_. When Ni is used, a transfer yield of more than 95% can be achieved, whereas Cu, Pd, and Au peel off graphene with difficulty. Due to the large difference between γ_Gr‐SiC_ and γ_Gr‐Gr_, there are still difficulties in directly separating single‐layer graphene from SiC with a Ni layer.

A single‐layer precision selective exfoliation method is developed according to the difference in binding energy between graphene and metals.^[^ ^84^ ^]^ As shown in Figure 3B, after exfoliating the epitaxial graphene layer from SiC using Ni as the first adhesive layer, Au film is deposited as the second adhesive layer to selectively remove the strips from the graphene sheet owing to γ_Gr‐Gr_ < γ_Gr‐Au_ < γ_Gr‐Ni_, leaving the monolayer graphene on the other side.

The above‐mentioned work is successful in obtaining large‐area graphene through metal‐assisted exfoliation but is limited to obtaining monolayer graphene grown on SiC substrates. A graphene exfoliation method with precise control of graphene thickness, named layer‐engineered exfoliation, is proposed.^[^ ^85^ ^]^ The random conventional exfoliation is transformed into a controllable and deterministic process by deposition of metal films using graphite as the material (Figure 3C). Different metals, such as Au, Pd, Ni, and Co, are directly evaporated onto a graphite flake as stress layers, which have different interfacial binding energies with graphene. As depicted in Figure 3E, graphene with various thicknesses can be obtained because of the difference in interfacial binding energy. The number of layers is determined by the applied torque's extension direction and the vertical crack's depth (Figure 3D). Cracks will form at the grain boundaries of graphene when an external bending moment is applied, propagating in parallel and normal directions. Parallel cracks influence the area of exfoliated graphene, while the depth of vertical crack determines the number of graphene layers, which increases with the rising of binding energy between graphene and metal. Metal films (Pd, Co, Ni) with higher binding energy are used to control the spalling depth to obtain large‐area multilayer graphene based on the proposed spalling mechanism (Figure 3E).

The exfoliation efficiency is affected by the type and thickness of metals, and the deposition method affects the quality of the resulting material. Choosing the right metal deposition method can help to reduce material damage and contamination while also increasing the exfoliation yield. Metals could be deposited directly on bulk materials, forming uniform and tight contact, but it may destroy the structure of materials and introduce considerable defects.^[^ ^92^, ^93^ ^]^ On the other hand, metal films could be deposited on an ultra‐flat substrate in advance, followed by attaching a thermal tape to make a metal tape, avoiding causing damage to the materials.^[^ ^89^ ^]^ To exfoliate the target material, the metal tape is pressed against the surface. However, it is easy to introduce contamination into this process, weakening the interaction between graphene and substrate and lowering exfoliation efficiency.

## 3 TAILORING OF GRAPHENE

To fabricate functional electronic devices, a variety of techniques have been used to tailor graphene.^[^ ^94^ ^]^ Traditional etching techniques in the semiconductor industry, such as photolithography and EBL, have made great breakthroughs with processing resolution improved to the nanoscale. Reactive ion etching (RIE), which is widely used in traditional etching processes, can also be applied to graphene tailoring. The basic working mechanism of RIE can be described as follows. Under the alternating electric field provided by radio frequency, the gas is excited and ionized, generating highly reactive plasma. The graphene basal plane is bombarded and reacted by ions and free radicals in the plasma. Particularly, physical etching reduces the etch selectivity, resulting in isotropic etching. For the preparation of graphene nanoribbons (GNRs) with a well‐controlled edge configuration, which is important for opening the band gap of graphene, atomic‐level etching is required.^[^ ^49^, ^95^, ^96^ ^]^ However, the required accuracy is still beyond the reach of current state‐of‐the‐art nanolithography techniques.

Pure chemical etching methods, such as reactive gas etching^[^ ^97^, ^98^, ^99^ ^]^ and remote plasma etching^[^ ^100^ ^]^, selectively remove C atoms at the edge of graphene owing to the difference in reaction rates between C atoms in zigzag and armchair sites, resulting in anisotropic etching with atomic precision. Metal nanoparticles, such as Ni^[^ ^101^ ^]^ and Fe,^[^ ^102^ ^]^ can anisotropically etch graphene when migrating on the basal plane, resulting in a crisscross line‐like etching pattern. However, this kind of etching occurs on the surface of graphene, where metal nanoparticles take random movements, making the etching sites uncontrollable, which cannot be applied to graphene patterning. After a brief introduction to EBL, we will primarily focus on anisotropic etching methods with various gases. The comparison of various anisotropic etching methods, in terms of etchant, substrate, edge configuration, and functional group, are summarized in Table 3.

**TABLE 3:** Comparison between different anisotropic etching methods

Etchant	Substrate	Edge configuration	Functional groups	Ref.
O_2_	SiO_2_/Si	Zigzag	Semiquinone	[97]
CO_2_	Cu foils	Zigzag	Lactone	[98]
H_2_O (gas)	SiO_2_/Si, Si_3_N_4_/Si, Mica	Armchair	Epoxy	[99]
H_2_ (plasma)	SiO_2_/Si	Zigzag	Hydrogen	[107]

### 3.1 Electron beam lithography

As a typical top‐down method for micro‐nano processing, EBL can achieve nanometer‐level resolution.^[^ ^103^ ^]^ Desired patterns can be drawn directly on the resist, then transferred by etching or evaporation to obtain patterned materials, based on the interaction between electrons and resist such as PMMA. For example, as a top‐down processing method, EBL in combination with oxygen plasma etching can obtain specifically patterned graphene.

**FIGURE 4:**
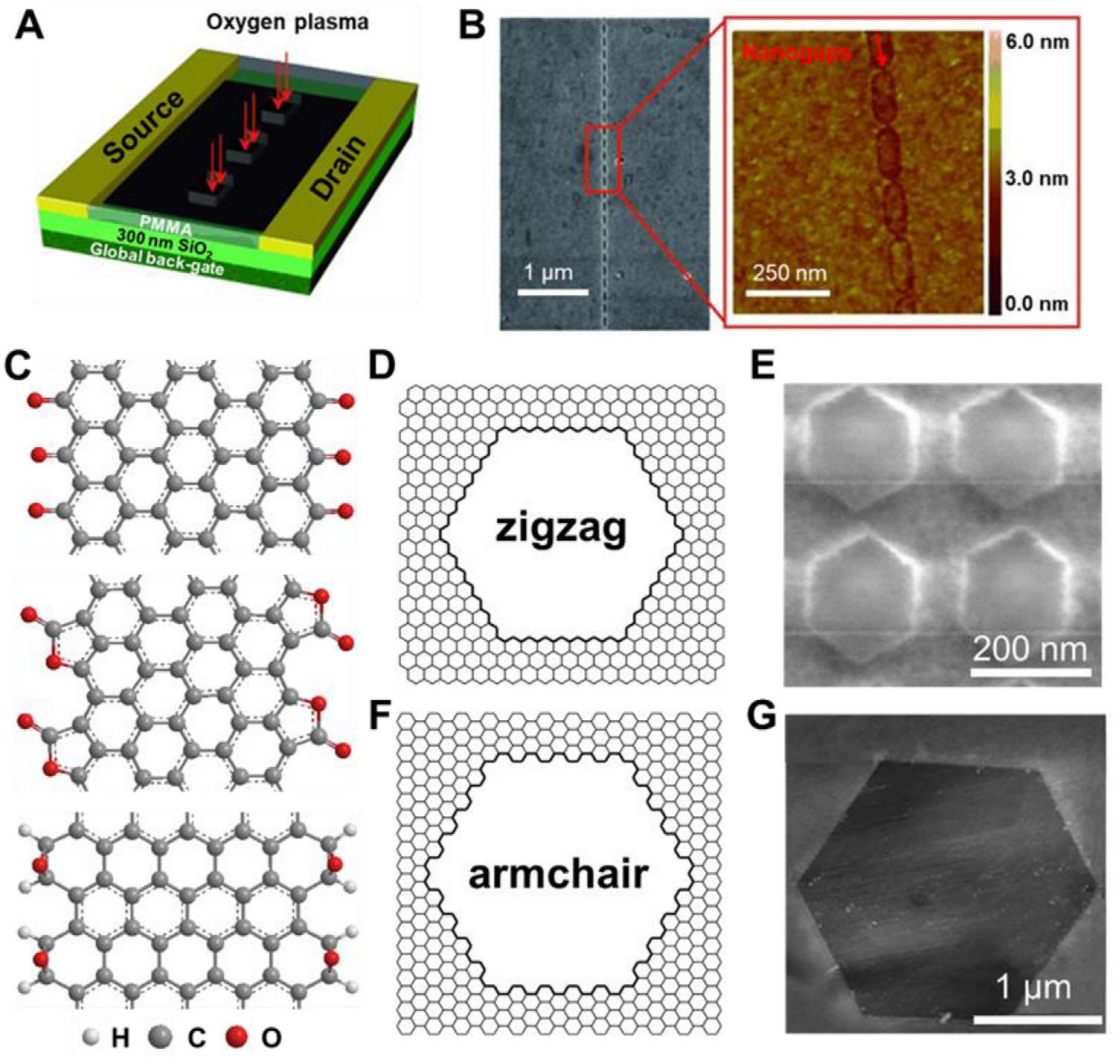
(A) Schematic of the tailoring of graphene with EBL and oxygen plasma etching. (B) SEM and AFM images of an achieved graphene point array. Reproduced with permission.^[^ ^104^ ^]^ Copyright 2012, Wiley‐VCH. (C) Vertical view of ZGNR terminated with carbonyl groups (top), ester groups (middle), and AGNR terminated with epoxy groups (bottom). (D,F) Schematic of the atomic structures of the etched hexagonal pits with zigzag and armchair edges. (E) SEM image of the O_2_‐etched hexagonal pits in graphene. Reproduced with permission.^[^ ^97^ ^]^ Copyright 2016, Wiley‐VCH. (G) AFM image of H_2_O‐etched hexagonal pit in graphene. Reproduced with permission.^[^ ^99^ ^]^ Copyright 2014, Wiley‐VCH

Due to its high resolution, EBL can prepare precise graphene nanopatterns, such as indented graphene point contact arrays.^[^ ^104^ ^]^ The graphene is cut through the window of PMMA by oxygen plasma etching after an indented window on PMMA is obtained by EBL (Figure 4A). By taking advantage of the PMMA's gradual etching and undercutting, narrow gaps (Figure 4B) can be obtained, as shown in the scanning electron microscope (SEM) and atomic force microscopy (AFM) images. However, the edges of the patterns obtained by EBL are not atomically regular, and the configuration of the edges has an influence on graphene‐based electrical devices.

### 3.2 Anisotropic etching of graphene

Traditional graphene nanostructure fabrication methods, such as EBL, have difficulty achieving atomic‐level precision edges. Chemical etching is commonly used in the top‐down methods.^[^ ^105^ ^]^ According to the difference in thermodynamic stability and reactivity between zigzag and armchair edges, some gases can be used as an etchant to achieve anisotropic etching.^[^ ^106^ ^]^ On the basal plane, hexagonal etching pits with controlled edges can be made in some cases.^[^ ^97^, ^98^, ^99^, ^107^ ^]^ Etching is influenced by temperature, etching time, substrate, and etchant concentration. By selecting a suitable etching condition, graphene with specific structures and edge configurations can be prepared.

#### 3.2.1 Anisotropic etching with O_2_

The oxidation reaction of carbon is the basis for graphene etching with oxygen.^[^ ^108^, ^109^ ^]^ EBL and RIE with oxygen‐plasma are used to pattern the initial holes on graphene as defects, and then low‐concentration O_2_ is introduced for further etching.^[^ ^97^ ^]^ On the basal plane, anisotropic etching of graphene can be achieved (Figure 4D,E). Following the Arrhenius‐law, the etching rate increase exponentially with temperature. The calculated reaction activation energy *E*_A_ is about 44 ± 4 kcal mol^−1^, which is equivalent to the activation energy of carbon oxidation reaction.^[^ ^110^, ^111^ ^]^ Based on the study of Raman D‐peak and weak localization, both of which are sensitive to intervalley scattering, the produced edges are confirmed to be predominantly of zigzag configuration.^[^ ^112^ ^]^ Temperature is considered to play a vital role in the oxidative anisotropic etching.^[^ ^113^ ^]^ The diminished difference in activation energy between the armchair and zigzag sites may cause a transition from hexagonal to circular pits at high temperature. A mechanism of graphene oxidation is proposed.^[^ ^110^, ^114^ ^]^ The chemisorption and dissociation of molecular O_2_ on the graphene edge account for the formation of zigzag edge terminated with semiquinone groups (Figure 4C, top).

Graphene has different etching characteristics on different substrates.^[^ ^115^ ^]^ After etching at 500°C for 2 h, graphene on mica and SiO_2_/Si shows obvious etched holes, while graphene on h‐BN is almost not etched. To study the influence of the substrate on the oxidative reactivity of graphene, the etching behaviors of graphene loaded on different substrates have been systematically studied. This phenomenon can be attributed to the differences in uniformity of charge distribution and topographic corrugations of substrates, both of which have effect on the reactivity of graphene loaded on its surface, resulting in different etching behaviors. Charged defects as well as the roughness of SiO_2_/Si both enhance the reactivity of graphene loaded on SiO_2_/Si. The surface of mica has the lowest roughness, but in presence of K^+^, the substrate has uneven charge distribution, resulting in etching sites besides the intrinsic defects of graphene. Although the surface of h‐BN is not as flat as mica's, the surface charge distribution is uniform, contributing to the substrate‐induced reactivity being relatively weak. The above results suggest that the enhanced reactivity is caused by the difference in charge distribution on the substrate rather than surface roughness, which can be regarded as a new strategy for selective modification of graphene.

#### 3.2.2 Anisotropic etching with CO_2_

Under the catalysis of copper foils, CO_2_ can also be used to etch single‐layer graphene grown by CVD.^[^ ^98^ ^]^ Anisotropic etching of graphene is observed when the CO_2_ flow rate is 5 sccm. More graphene is etched away as the rate of CO_2_ flow increases. The 120° angle between the adjacent edges of graphene can be clearly observed, suggesting the anisotropic etching with CO_2_. CO_2_ chemisorption on carbonaceous surfaces is investigated theoretically, providing clues for determining edge functional groups after etching.^[^ ^116^ ^]^ The heats of CO_2_ adsorption are significantly different for the formation of different functional groups. Lactone carbon–oxygen group formation at the zigzag edge is the most exothermic reaction, according to the calculations, implying the possibility of zigzag edges terminating with lactone groups (Figure 4C, middle). Therefore, CO_2_ etching also provides a new strategy for the fabrication of graphene nanostructures.

#### 3.2.3 Anisotropic etching with H_2_O

According to the water–gas reaction, water is an effective etchant for graphitic material.^[^ ^117^, ^118^, ^119^ ^]^ Therefore, water vapor is also used as an etchant for anisotropic graphene etching.^[^ ^99^ ^]^ Different from O_2_ etching and CO_2_ etching, water vapor etching can produce a regular armchair edge (Figure 4F,G). Specifically, graphene has been placed on SiO_2_/Si, Si_3_N_4_/Si, and mica by mechanical exfoliation, and then etched at 850−100°C in water vapor introduced by argon gas flow. After etching, uniform‐orientated hexagonal holes with armchair edges can be obtained. Most oxygen‐containing functional groups, with the exception of epoxy groups, cannot exist at very high temperatures. Therefore, the armchair edge is ended with epoxy groups above 950°C. According to the calculated binding energy of zigzag graphene nanoribbons (ZGNRs) and armchair graphene nanoribbons (AGNRs) terminated with epoxy oxygen atoms, the epoxy‐terminated armchair edge is more stable than the zigzag edge, which explains the existence of the armchair edge (Figure 4C, bottom). Therefore, water vapor etching allows for more precise control of the competitive etching of the edge configuration, resulting in graphene edges with an armchair configuration.

#### 3.2.4 Anisotropic etching with H_2_

**FIGURE 5:**
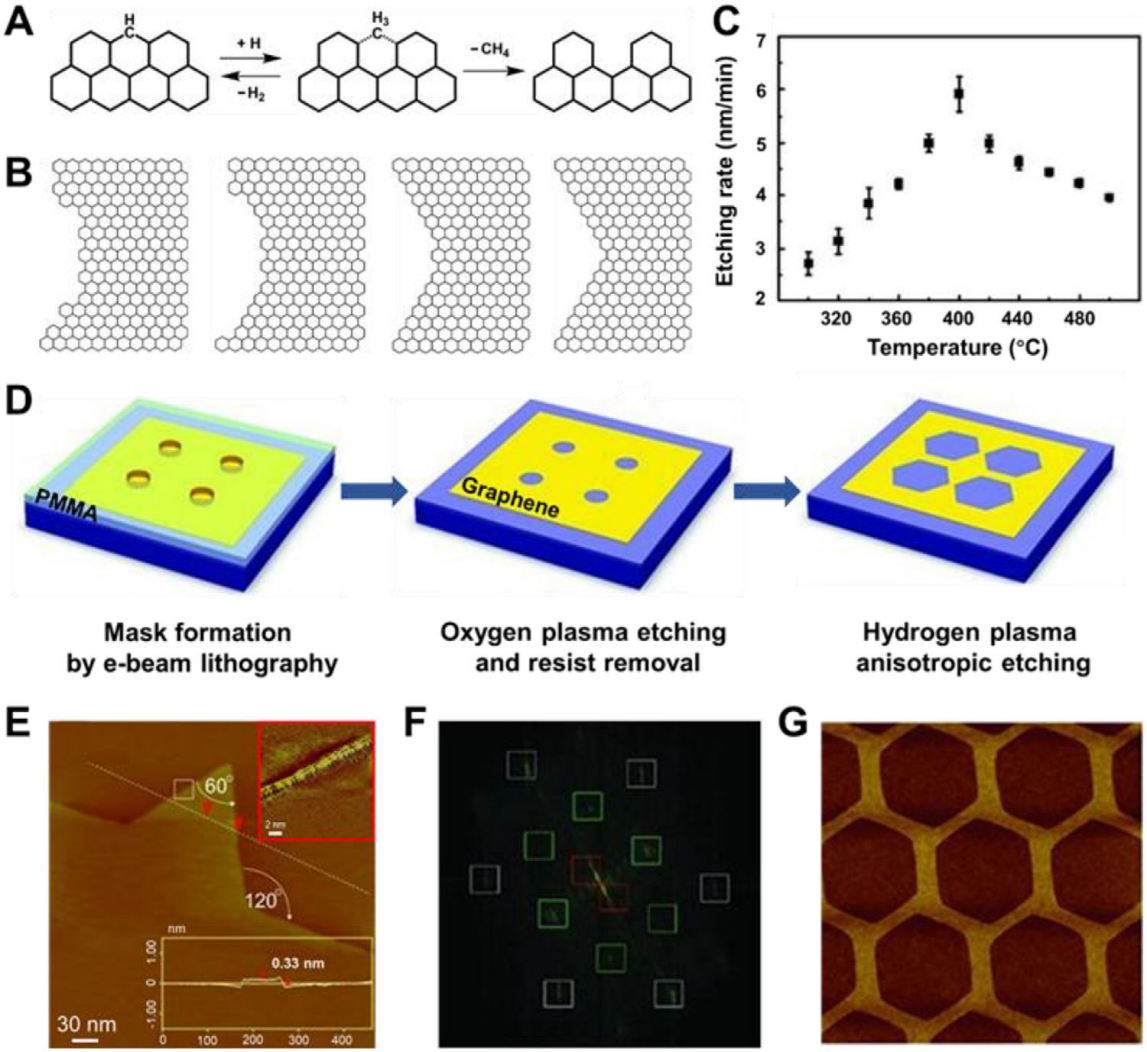
(A) Processes of the etching reaction between hydrogen atoms and graphene edges. (B) Schematic diagram of the evolution of graphene edges from round to hexagonal pits. (C) Temperature‐dependent etching rates for trilayer graphene with hydrogen plasma. Reproduced with permission.^[^ ^100^ ^]^ Copyright 2018, Springer. (D) Schematic of multi‐step processing of graphene with EBL, oxygen‐plasma, and anisotropic hydrogen plasma etching. Reproduced with permission.^[^ ^120^ ^]^ Copyright 2011, Wiley‐VCH. (E) Scanning tunneling microscopy image of a monolayer graphene with hexagonal pit. Inset shows atomic resolution image of the edge. (F) The Fourier transform spectrum (power spectrum) of the corresponding sample. Reproduced with permission.^[^ ^107^ ^]^ Copyright 2010, Wiley‐VCH. (G) Honeycomb‐like graphene networks produced with hydrogen plasma etching. Reproduced with permission.^[^ ^120^ ^]^ Copyright 2011, Wiley‐VCH

In addition to oxidative etching, hydrogen plasma also has a significant anisotropic etching effect on graphene (Figure 5A,B), which can be regarded as an effective method to produce graphene edge with a specific zigzag configuration (Figure 5E,F).^[^ ^107^ ^]^ Hexagonal etching holes of various sizes are randomly distributed with uniform orientations on the graphene surface after etching with hydrogen plasma. The number of hexagonal etching holes on the surface of few‐layer graphene is dramatically reduced compared to single‐layer graphene. From the results of Raman spectroscopy, it can be observed that the basal plane remains intact without defects, indicating the graphene still has high‐quality after etching. The binding energy of ZGNR with hydrogen is reported to be obviously higher than that of AGNR,^[^ ^99^ ^]^ reflected in the better stability of hydrogen‐terminated zigzag edges, which is in good agreement with the experimental results. Hydrogen plasma can be considered an ideal tool for graphene tailoring because of its controllable etching rate and ability to maintain basal plane integrity.

Furthermore, artificial round defects can be created on the basal plane of graphene via EBL and oxygen‐plasma etching to induce plasma etching reactions.^[^ ^120^ ^]^ On the basal plane, a uniformly oriented hexagonal etching hole array can be produced after hydrogen plasma etching (Figure 5D). Different nanostructures (Figure 5G), such as graphene superlattices, triangular islands, and ZGNRs, can be obtained by changing the original direction of hole arrangement, etching rate, and time. Utilizing the precise patterning ability of EBL and the controllability of hydrogen plasma etching, it provides a promising approach for the fabrication of graphene devices and circuits.

The origin of the anisotropic etching effect in remote hydrogen plasma etching is revealed, and its dependence on the substrate, temperature, and number of layers is explained, using a geometrical model.^[^ ^100^ ^]^ For single‐layer graphene, the roughness and surface charges of the SiO_2_/Si surface heavily decrease the etching barrier of both zigzag and armchair edges. Isotropic etching is caused by a decrease in the difference in reactivity between the two configurations. The intrinsic difference in reactivity between zigzag and armchair edges is preserved in multilayer graphene due to the passivation from the bottom‐layer graphene, resulting in anisotropic etching. The etching process can be divided into three procedures (Figure 5A). Firstly, hydrogen atoms are adsorbed at the graphene edge, accompanied by the configuration of edge carbon atoms changing from sp^2^ hybridization to sp^3^ hybridization. Then, the C─C bonds are broken under the attack of hydrogen atoms, which is rate‐limiting due to the large reaction barrier. Finally, the unstable ─CH_3_ group is hydrogenated to form CH_4_. Only at a moderate temperature (400°C) can the hydrogenation of C atoms at the edge and the breakage of the C─C bonds be satisfied at the same time. Therefore, the reaction rate takes a “volcano type” curve with temperature changes (Figure 5C). The shape evolution of graphene pits from circular to hexagonal can be described using kinetic Wulff construction.^[^ ^121^ ^]^ Due to the better stability and the slower etching rate of zigzag edges, other edge configurations would gradually transform to the zigzag configurations, contributing to the hexagonal etching hole with zigzag edge‐configuration (Figure 5B).

## 4 MODIFICATION OF GRAPHENE

Broadly speaking, modification of graphene involves noncovalent functionalization and covalent functionalization. Noncovalent functionalization makes physical contact with graphene in order to alter its electrical properties while causing minimal damage to the basal plane.^[^ ^33^, ^34^ ^]^ At the same time, the covalent modification of sp^2^ carbon materials has previously been extensively applied to graphene, fullerenes, and carbon nanotubes.^[^ ^122^ ^]^ Because these structures consist of benzene, organic reactions about benzene are extended to fullerene chemistry and graphene chemistry, such as Friedel–Crafts acylation, Diels–Alder cycloaddition, nitrene addition, Claisen rearrangement, and 1,3‐dipolar cycloaddition. In general, graphene chemical functionalization involves basal‐plane and edge modification. Most basal‐plane modifications are about the rehybridization of sp^2^ to sp^3^ carbons, while most edge modifications occur at the defect sites of graphene.^[^ ^123^, ^124^, ^125^ ^]^ The chemical functionalization of graphene improves the optical and electrical properties of graphene‐based materials, as well as their solubility.^[^ ^126^, ^127^, ^128^ ^]^ Functionalized graphene can also be incorporated into composite materials for optoelectronic applications.^[^ ^129^, ^130^, ^131^ ^]^

### 4.1 Noncovalent functionalization

Noncovalent functionalization by π–π interaction is a promising approach to attaching functional groups to graphene without destroying the conjugated framework.^[^ ^132^, ^133^, ^134^ ^]^ There are five types of noncovalent interactions: cation‐type interactions, anion‐type interactions, hydrogen bonding, hydrophilic/hydrophobic interactions, and π–π stacking interactions.^[^ ^135^ ^]^ π–π stacking interactions are relative to the fabrication of nanodevices and building sensing devices of graphene.^[^ ^136^ ^]^ Electron–electron interaction can tune the electron density of graphene by π–π stacking.^[^ ^137^, ^138^ ^]^

Because of its strong interactions with the basal plane of graphene through π–π stacking, the pyrene moiety is typically used as a noncovalent functional group.^[^ ^139^, ^140^ ^]^ Other groups can also be added to realize a wide range of applications based on the pyrene moiety.^[^ ^141^, ^142^ ^]^ Modification of graphene with pyrene‐modified species is intrinsically limited to sub‐monolayer or monolayer coverage. Noncovalent approaches to graphene modification, on the other hand, have the disadvantages of instability and poor reproducibility.^[^ ^135^ ^]^

### 4.2 Covalent functionalization

Covalent functionalization allows for stable bonding between graphene and functional groups, which could be important for optoelectronic devices operation with improved stability.^[^ ^143^ ^]^ In the following section, we aim to give a timely survey about major strategies for covalent functionalization of graphene, including electrophilic substitution reaction, free radical reaction, and cycloaddition reaction.

#### 4.2.1 Electrophilic substitution reaction

An electrophilic reagent attacks the negative electron system to replace the weaker electrophilic group in an electrophilic substitution reaction, which is a common type in aromatic systems. The electrophilic reagent attacks the edges of graphene without damaging the basal plane, and the types include Friedel–Crafts acylation, chlorination, and sulfonation.

##### Friedel–Crafts acylation

As a typical electrophilic aromatic substitution reaction, Friedel–Crafts acylation reaction allows the synthesis of acylated products from the reaction between arenes and electrophiles. The edge‐selective graphene modification in a mild polyphosphoric acid (PPA)/phosphorous pentoxide (P_2_O_5_) medium can be achieved at ∼130°C using the “direct” Friedel–Crafts acylation.^[^ ^37^ ^]^ The reactive C═O^+^ is generated by protonation of a carbonyl group of the benzoic acid derivatives via PPA.^[^ ^48^ ^]^ With the benzoic acid or benzamide derivatives, any desired functional groups, such as small molecules or macromolecules, can be attached to the edges of graphene.^[^ ^144^, ^145^ ^]^ The C═O^+^ ion attacks the edges/defects of graphene without destroying the basal plane to preserve the conductivity and integrity of graphene in a mild and controllable way.^[^ ^37^ ^]^ Various functional wedges are introduced to the edge of graphite, endowing graphene materials with specific functions.

**FIGURE 6:**
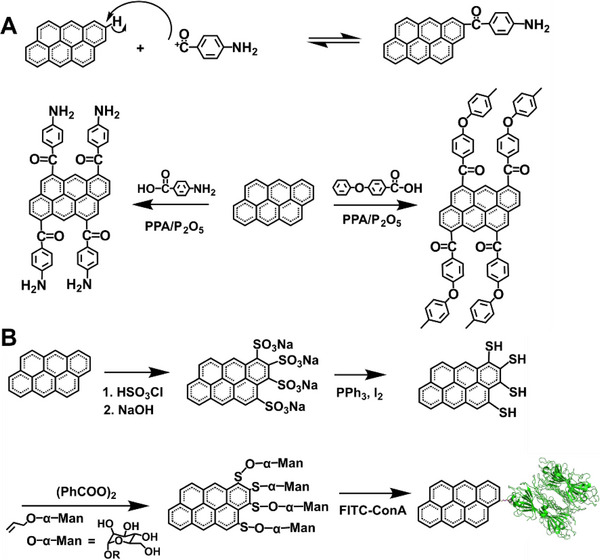
(A) Reaction mechanism of the direct Friedel–Craft acylation reaction (top). Reaction between graphite and 4‐aminobenzoic acid or *para*‐poly(ether‐ketone) via the Friedel–Craft acylation (down). Reproduced with permission.^[^ ^144^, ^146^ ^]^ Copyright 2010, The Royal Society of Chemistry. (B) Synthesis of graphene sulfonate by electrophilic substitution. Reproduced with permission.^[^ ^38^ ^]^ Copyright 2018, Springer Nature

Small molecules, linear and hyperbranched macromolecules, are attached to the edges of graphene, as illustrated in Figure 6A. Edge‐selective functionalization of graphene with small molecules, such as 4‐aminobenzoic acid (ABA), was carried out for the first time in 2010.^[^ ^144^ ^]^ The amount of ABA in edge‐functionalized graphene (EFG) is determined by thermogravimetric analysis (TGA) showing ∼36 wt%. The covalent attachment of ABA grafts at the graphene edges has been verified using AFM images. The Friedel‐Crafts acylation reaction can introduce linear and hyperbranched polymers onto the edges of graphitic layers.^[^ ^146^, ^147^ ^]^ For example, the para‐poly (ether‐ketone) (pPEK) can be covalently grafted to graphite.^[^ ^146^ ^]^ A series of characterization can verify the success of modification. The resulting pPEK grafted graphite (LPEK‐g‐graphene) shows better tensile properties than pPEK films. Dendritic macromolecular wedges can also be directly grafted to the edges of graphite via the Friedel–Crafts acylation. The solubility of dendritic macromolecules is improved in comparison with that of linear analogs. In general, graphite is treated with AB_2_ monomer for hyperbranched poly(ether ketone) (HPEK).^[^ ^147^ ^]^ According to AFM images, the edge of graphene is higher than its basal plane due to the existence of HPEK. The high degree of exfoliation is estimated using wide‐angle X‐ray diffraction. The cyclic voltammetry experiments results show that the carboxylic acid groups exist. The above‐mentioned aromatic 4‐aminobenzoyl moieties on the edge of AB‐EFG can play a dual role in “C‐welding” and “N‐doping” to obtain N‐graphite films, which have high conductivity, good mechanical properties, and high optical transmittance.^[^ ^148^ ^]^ N‐doped graphene has tremendous potential applications for its superior performance in field‐effect transistors (FETs).^[^ ^149^, ^150^ ^]^

##### Friedel‐Crafts chlorination

A Friedel‐Crafts chlorination reaction for edge chlorination of nanographene is reported as a vital step toward controlled functionalization, resulting in well‐defined chemical functionalized graphene materials.^[^ ^151^ ^]^ In comparison with the graphene modification mentioned above, Friedel–Crafts chlorination happens at the edges/defects of graphene as confirmed by X‐ray single‐crystal analysis, which benefited from the solubility of nanographene to obtain the crystal structure. A series of well‐defined nanographene (C_42_H_18_, C_48_H_18_, C_60_H_22_, C_60_H_24_, C_96_H_30_, C_132_H_34_, and C_222_H_42_) are chosen to reflect precise chlorination. Chlorination is achieved at ∼80°C in CCl_4_ with iodine monochloride and catalyzed by AlCl_3_. Matrix‐assisted laser desorption/ionization‐time of flight mass spectrometry confirms the elemental composition of chlorinated products. The isotopic distribution pattern of the mass peak is in good agreement with the calculated pattern. The precise edge chlorination can be served as an efficient model for edge‐functionalized graphene materials, providing an efficient way to tune properties of nanographene such as solubility, band gap, and the position of the frontier molecular orbital.

##### Electrophilic sulfonation

Unlike the Friedel–Crafts acylation, carbonyl groups cannot be introduced by the electrophilic aromatic substitution, which could slow electron transfer between the basal plane and the functional groups. Sulfur is electrophilic to attack electron‐rich graphene, then graphene sulfonate (G─SO_3_^‒^) is prepared by chlorosulfonation and finally reduced to form graphene thiol (G─SH) (Figure 6B).^[^ ^38^ ^]^ An edge‐modified graphene bioconjugate is synthesized through the radical addition of allyl mannoside. The modified graphene has different solubilities, which can be dispersible in toluene. The X‐ray photoelectron spectroscopy (XPS) of sulfur‐modified samples shows the standard signatures for both sulfonate and thiolate groups, and the S:C atomic ratio of G─SO_3_^−^ and G─SH is analyzed to be ∼0.3:100. Chemical functionalization of edges and defect sites of basal plane are visible in fluorescent‐labeled graphene under super‐resolution microscopy. It is possible to create the “glycographene” by radical addition, demonstrating the synthetic feasibility of directly linking reaction groups. Edge localization is visualized by epifluorescence microscopy of a lectin‐glycographene bioconjugate with fluorescently tagged.

Electrophilic sulfonation is also realized by edge‐selective functionalization with chlorosulfuric acid.^[^ ^152^ ^]^ In this reaction, generated electrophilic ions overcome 2D π–π stacking of graphite and are covalently introduced into the edge of graphite, which ultimately turns to the more stable sulfonated graphene. SEM images of graphite have a flake shape with smooth surfaces, while sulfonated graphene shows an aggregated morphology indicating the presence of H‐bonds among polar sulfonic acid molecules at the graphene edges. The transmission electron microscopy (TEM) images indicate no chemical damage to the basal plane. Graphite can have up to ∼22.5 wt% edge‐selective functionalization.

#### 4.2.2 Free radical reaction

Chemical reactions, laser irradiation, and photochemistry can all produce free radicals. Using diazonium salts, benzoyl peroxide, and halogen, free radicals can be added to the basal plane, transforming sp^2^ carbons to sp^3^ carbons.

##### Diazonium reaction

**FIGURE 7:**
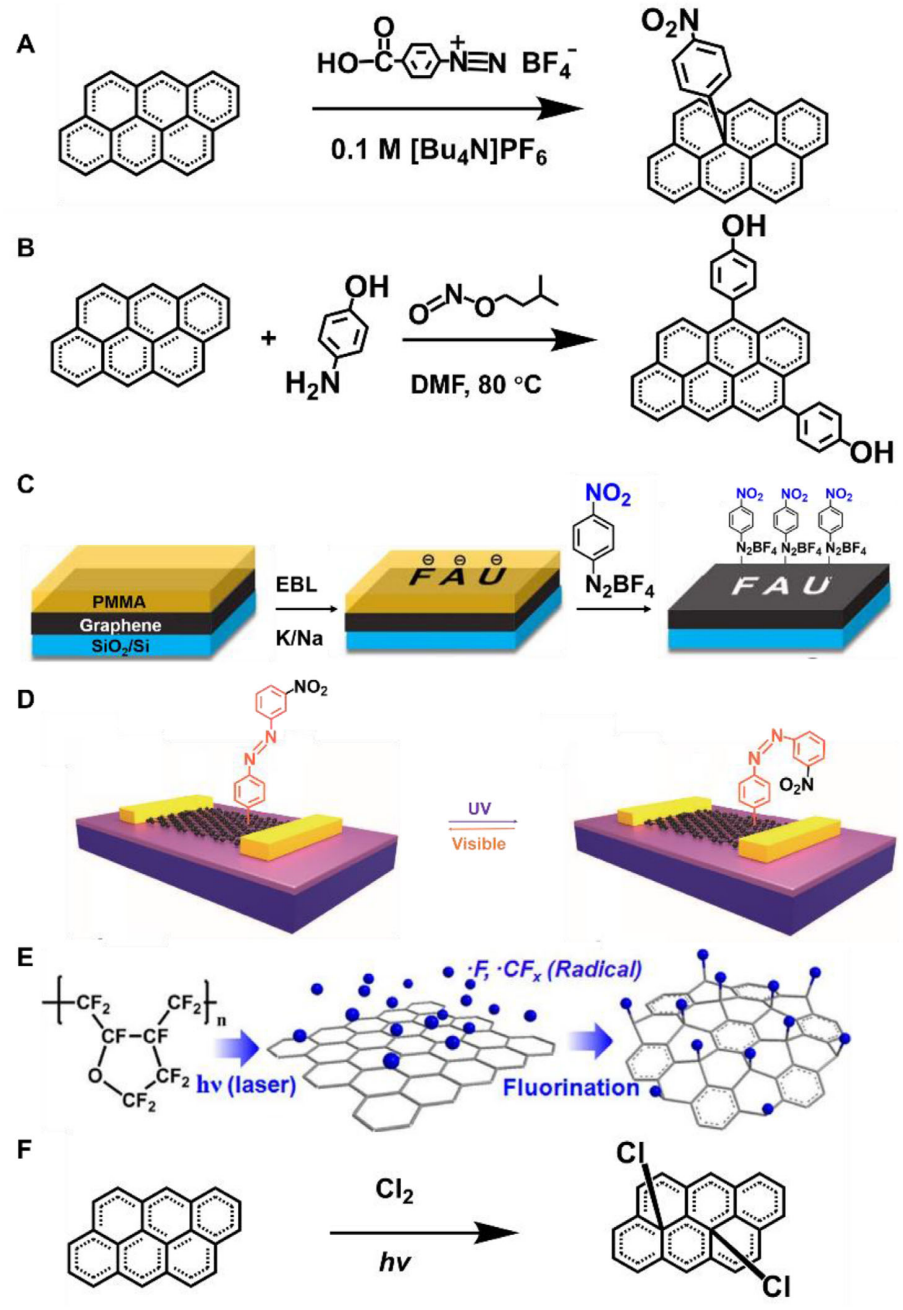
(A) Attaching aryl groups to graphene by means of reduction of 4‐nitrophenyl diazonium tetrafluoroborate. Reproduced with permission.^[^ ^153^ ^]^ Copyright 2009, American Chemical Society. (B) Edge‐selective functionalization of graphene with 4‐aminophenol. Reproduced with permission.^[^ ^157^ ^]^ Copyright 2018, Wiley‐VCH. (C) Schematic illustration of the reaction sequence for the patterned functionalization of graphene. Reproduced with permission.^[^ ^155^ ^]^ Copyright 2019, Wiley‐VCH. (D) Functionalization and optical switching of graphene devices induced by isomerization of the azo group. Reproduced with permission.^[^ ^159^ ^]^ Copyright 2018, Wiley‐VCH. (E) Mechanism for fluorination by using fluoropolymer and laser irradiation. Reproduced with permission.^[^ ^160^ ^]^ Copyright 2012, American Chemical Society. (F) The photochemical chlorination process of graphene. Reproduced with permission.^[^ ^161^ ^]^ Copyright 2011, American Chemical Society

Diazonium reaction is one of the most vital types of free radical reactions. Surface modification of graphene with nitrophenyl groups is accomplished through the spontaneous reaction of the diazonium salt (Figure 7A).^[^ ^39^, ^153^, ^154^ ^]^ Delocalized electrons are transferred from graphene to aryl diazonium cations, which produce aryl groups of graphene after releasing a molecule of N_2_. The successful attachment of C_6_H_4_─NO_2_ groups on the graphene sheet can be confirmed by the Fourier transform infrared spectroscopy (FTIR) and time‐of‐flight secondary ion mass spectrometry (TOF‐SIMS) characterizations. The modification of the graphene largely enhances the room temperature resistance and changes the transport properties and electronic structure of graphene. For instance, modified graphene possesses a ∼0.4 eV bandgap. Using diazonium functionalization of graphene, it is possible to modify graphene not protected by PMMA to realize patterning and embroidery.^[^ ^155^ ^]^ Regular multiply functionalized patterns form molecular graphene embroidery including concentric regions of covalent addend binding.^[^ ^156^ ^]^ Furthermore, diazonium reaction and thermal treatment to functionalize the spatial structure using EBL on PMMA have been used to achieve reversible covalent patterning of graphene (Figure 7C).^[^ ^155^ ^]^

Diazonium chemistry can also be used to synthesize the edge‐selective functional graphene nanoplatelets (GnP) (Figure 7B).^[^ ^157^ ^]^ The dianiline‐bridged GnP constitutes molecular junctions that maintain a defect‐free sp^2^ structure. Chemical functionalization can drive nanoflakes to self‐organize into nanopaper, which has excellent thermal conductivity. The arylation of graphene can also be realized using diazonium salts by arylazocarboxylic tert‐butyl esters.^[^ ^158^ ^]^ In situ covalent functionalization of graphene devices can realize the photoswitching function with photochromic azobenzene moieties using diazonium chemistry (Figure 7D). Illumination with UV and visible lights can change the conformation of azobenzene, resulting in a reversible optically tunable change in a graphene doping level.^[^ ^159^ ^]^

##### Fluorination reaction

Several techniques have been used to fluorinate sp^2^ carbons, such as exposure to F_2_ gas at moderate temperatures (400−600°C) and treatment with F‐based plasma.^[^ ^40^ ^]^ Graphene can be fluorinated with xenon difluoride (XeF_2_) to form perfluorographane. The single‐sided exposure can achieve ∼25% coverage of fluorination saturation (C_4_F). The fluorinated graphene has insulating properties. In the case of C_4_F, the bandgap is ∼2.93 eV, and its π systems are surrounded by sp^3^ C atoms. In the other case, graphene is covered by fluoropolymer and irradiated with laser to generate fluorinated graphene (Figure 7E).^[^ ^160^ ^]^ Only the laser‐irradiated region undergoes the reaction. Fluorinated graphene with highly insulating properties is produced by laser irradiation of fluoropolymer‐covered graphene.

##### Photochemical chlorination

The photochemical chlorination of graphene leads to the structural transformation of C─C bonds to open up a bandgap of graphene (Figure 7F).^[^ ^161^ ^]^ In a free radical addition reaction, the chlorine radicals can be formed by relative molecules. After the prepared radicals react with the graphene, the chlorine is grafted onto the graphene's basal plane, resulting in a homogeneous and nondestructive photochlorinated graphene. After photochlorination, the room‐temperature conductance of graphene drops by three orders of magnitude. The exposed region can be efficiently photochlorinated, where the masked regions would be used for all‐graphene circuits.

#### 4.2.3 Cycloaddition reaction

Cycloaddition reaction is another important organic reaction for graphene modification, in which the terminal carbon atoms of two conjugated systems are connected head to tail to form two *σ* bonds. Thus, 1,3‐dipolar cycloaddition, Bergman cycloaddition, Diels–Alder reaction and nitrene addition are used to combine the two molecules into a larger cyclic molecule.

##### 1,3‐dipolar cycloaddition

**FIGURE 8:**
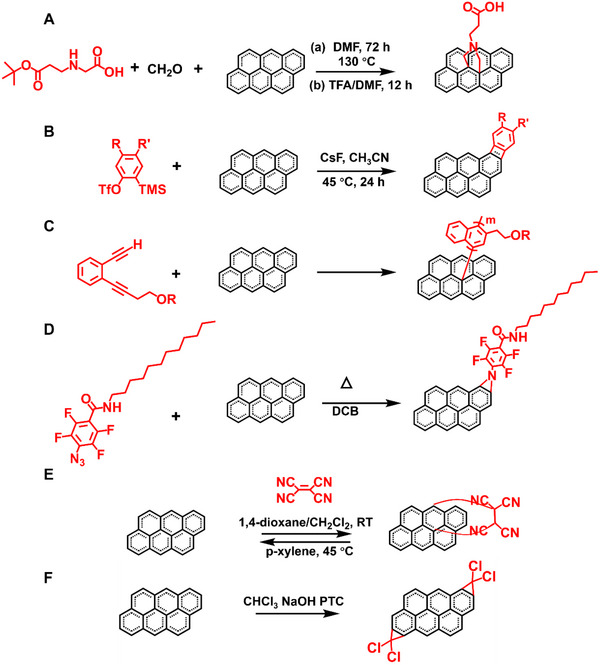
(A) Cycloaddition of graphene by condensation of paraformaldehyde with a modified α‐amino acid. Reproduced with permission.^[^ ^162^ ^]^ Copyright 2011, The Royal Society of Chemistry. (B) Schematic of aryne cycloaddition of graphene. Reproduced with permission.^[^ ^163^ ^]^ Copyright 2010, The Royal Society of Chemistry. (C) Functionalization of graphene with enediyne molecules. Reproduced with permission.^[^ ^164^ ^]^ Copyright 2012, Wiley‐VCH. (D) Functionalization of graphene with PFPA. Reproduced with permission.^[^ ^166^ ^]^ Copyright 2010, American Chemical Society. (E) D‐A reaction between graphene (diene) and tetracyanoethylene (TCNE, dienophile). Reproduced with permission.^[^ ^167^ ^]^ Copyright 2011, American Chemical Society. (F) Functionalization of graphene via dichlorocarbene cycloaddition reaction. Reproduced with permission.^[^ ^169^ ^]^ Copyright 2012, The Royal Society of Chemistry

Using 1,3‐dipolar cycloaddition, graphite can be successfully functionalized and exfoliated.^[^ ^41^, ^42^, ^162^ ^]^ The amino‐functionalized graphene can be attached to gold nanorods (AuNRs), which can be used to locate the reactive sites of graphene (Figure 8A).^[^ ^41^ ^]^ UV–vis spectroscopy and TEM characterizations confirm the interaction between AuNRs and functionalized graphene. Macromolecules like poly(amidoamine) dendron can also be used to functionalize graphene. It is found that the graphene is not only functionalized at the edges, but on the surface as well. Both the 1,3‐dipolar cycloaddition and the amide‐bond condensation reaction on the prepared carboxylic groups can be generated by sonicating process.^[^ ^162^ ^]^ It has also been accomplished to make superior pyrrolidine‐functionalized graphene sheets which is single‐ or few‐layer with fluorine contained.^[^ ^42^ ^]^ Graphite is treated with sarcosine and pentafluorobenzaldehyde via 1,3‐dipolar cycloaddition to obtain certain graphite with the surface modified.

##### Aryne cycloaddition

The aryne cycloaddition of graphene can be realized under mild reaction conditions. The aryne‐modified graphene sheets are stable and dispersible in a variety of solvents (Figure 8B).^[^ ^163^ ^]^ F can be further covalently modified on graphene by treating it with the F‐substituted benzyne species, as shown by XPS and FTIR characterizations. Therefore, aryne cycloaddition is a facile and mild approach to obtain aryne functionalized graphene with good dispersibility.

##### Bergman cyclization

Bergman cyclization is a cycloaromatization of enediyne‐containing compounds carried out a diradical intermediate (Figure 8C).^[^ ^164^ ^]^ Various enediynes directly attack sp^2^ carbon atoms at the surface of graphene. The modified graphene is soluble in many kinds of organic solvents and has excellent electric conductivity. This unique self‐coupling method can also be used to make polymer‐grafted graphite.

##### Nitrene addition

Graphene nanosheets can be functionalized through nitrene addition of azido‐phenylalanine to exfoliate graphite (Figure 8D).^[^ ^165^ ^]^ The nitrene addition could not take place only at the sheet's edges, and the resulting structure is similar to graphene oxide. The other method of nitrene addition is photochemical or thermal activation of graphene with perfluorophenyl azide (PFPA), which is converted to the active singlet perfluorophenylnitrene and undergoes C═C addition reactions with the graphene to produce the aziridine adduct.^[^ ^166^ ^]^ The solubility of graphene can be greatly regulated by chemical functionalization, and the functionalized graphene products disperse well in the corresponding solvents.

##### Diels–Alder reaction

Through the Diels–Alder (D‐A) reaction, graphene can be used as diene or dienophile to modify the electronic properties under mild conditions (Figure 8E).^[^ ^167^ ^]^ Specifically, graphite and graphene are used as dienophiles, both the 9‐methylanthracene and the 2,3‐dimethoxy‐1,3‐butadiene are used as dienes for the D‐A reaction. The carbons in graphene manage to transform from sp^2^ hybridized to sp^3^ hybridized through the D‐A chemistry. Attenuated total reflectance FTIR spectroscopy can be used to confirm the generality of graphene D‐A reactions. A solvent‐free D‐A reaction has been carried out by heating a mixture of graphite and typical dienophiles, such as maleic anhydride (MA) and maleimide (MI).^[^ ^168^ ^]^ Various characterization techniques have confirmed the functionalization of graphite with dienophiles, implying that graphite can be functionalized and delaminated into a few layers of graphitic nanosheets efficiently.

##### Dichlorocarbene functionalization

Since carbene functionalization on carbon nanotubes has been studied extensively, it is possible that dichlorocarbene functionalization on graphene will occur naturally (Figure 8F).^[^ ^169^ ^]^ The addition dichlorocarbene, diarylcarbene, and fluorinated olefins is the focus of most experimental work. The functionalization is operated based on a conventional method which is used to produce the dichlorocarbene. In brief, the graphene is suspended in a mixed solution of chloroform (CHCl_3_), sodium hydroxide (NaOH), and phase transfer catalyst under reflux.

#### 4.2.4 Reaction with small molecules

Mechanochemical methods and microwave assisted methods can cause some small gas molecules to react on the edge of graphene. This method is simple, low‐cost, environmentally friendly, and scalable for mass‐producing graphene nanoplatelets (GnPs) with various functional groups.

##### Mechanochemical assisted reaction

A variety of functional groups could be grafted to the edge of graphite when mixing the graphene with solids, liquids, or even chemical vapors in a ball‐mill crusher according to the availability of mechanochemical reactions driven by ball milling. During ball milling, the high‐speed rotation of the stainless‐steel balls generates sufficient kinetic energy to break the graphitic C─C framework, and then various functional groups can be introduced at the broken edges of graphite. Edge‐selective functionalized graphene nanoplatelets can be achieved through mechanochemical reactions with hydrogen, carbon dioxide, sulfur trioxide, or carbon dioxide/sulfur trioxide mixture. The gas is captured by mechanochemically produced active carbon species resulting in graphene with edge‐selective functionalization.

**FIGURE 9:**
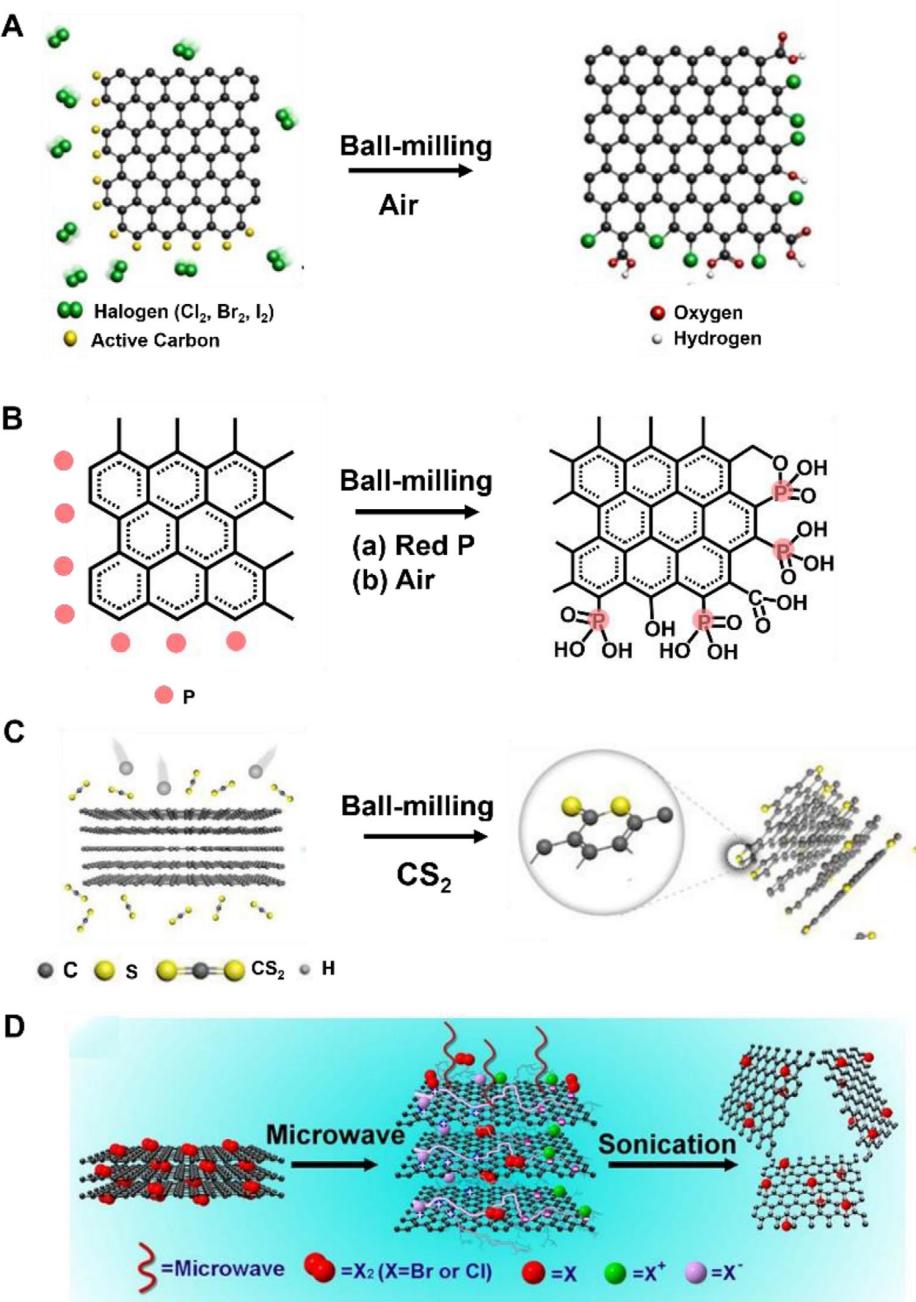
(A) A mechanochemically induced edge‐halogenation reaction between active carbon species and reactant halogens. Reproduced with permission.^[^ ^170^ ^]^ Copyright 2013, Springer Nature. (B) Mechanochemical functionalization of graphene in the presence of red phosphorus. Reproduced with permission.^[^ ^172^ ^]^ Copyright 2014, American Chemical Society. (C) Mechanochemical synthesis of TAGnPs in the presence of CS_2_. Reproduced with permission.^[^ ^174^ ^]^ Copyright 2019, Elsevier Ltd. (D) The halogen reacting with the graphite with the assistance of microwave irradiation. Reproduced with permission.^[^ ^175^ ^]^ Copyright 2012, Elsevier Ltd

A series of edge‐selective halogenated graphene nanoplatelets (XGnPs = ClGnP, BrGnP, and IGnPs) have been synthesized by mechanochemical reactions in the presence of a halogen gas (Figure 9A).^[^ ^170^ ^]^ Active carbon species are formed when the edges of graphite are broken, which are reactive to pick up halogens. The presence of Cl, Br, and I is successfully confirmed by energy dispersive X‐ray spectroscopy with element mapping. Furthermore, it is found that the edge‐halogens cause the edge expansion of XGnPs. Two‐step ball‐milling of graphene yields the graphene nanoplatelets (ISGnPs) with iodine/sulfonic acid functionalized at the edge.^[^ ^171^ ^]^ First, graphite can be edge functionalized by iodine under ball‐mining to produce IGnPs, followed by ball‐milling with sulfur trioxide to produce ISGnPs. Phosphorus is covalently attached to the edges of graphene nanoplatelets by ball‐milling (Figure 9B).^[^ ^172^ ^]^ During the mechanochemical ball‐milling process, C─C bonds are cleaved into activated carbon species, reacting with phosphorus to form graphene phosphorus. The mechanochemical ball‐milling time can be adjusted to control element content and gain size. Graphene is violently oxidized to form phosphonic acid when moisture is introduced further. At the atmosphere of dry ice, the edge‐selective carboxylated graphite (ECG) is obtained with high yield by ball milling.^[^ ^173^ ^]^ The generated ECG can be dispersed in a lot of polar solvents to be exfoliated into graphene nanosheets. Because of the hydration induced by ball‐milling, the high‐energy carboxylates turn into carboxylic acids (─COOH), and residual activated carbon species turn into hydroxyl (─OH) and hydroperoxyl (─OOH) by oxygen and moisture in the air. The ball milling process makes the surface area increase to large extents. Carbon disulfide (CS_2_) can also be introduced to the edges of GnPs with abundant ─C═S/─C─S bonds through mechanochemical reactions with CS_2_ (Figure 9C). The resultant edge‐thionic acid‐functionalized GnPs (TAGnPs) retain a similar structure of pristine graphite.^[^ ^174^ ^]^ The ─C═S/─C─S bonds at the edges of TAGnPs enable stronger Li^+^ adsorption capability, which can be used for high‐performance lithium‐ion batteries.

Mechanochemical reactions with hydrogen, sulfur trioxide, carbon dioxide, or a mixture of sulfur dioxide and carbon trioxide can efficiently produce the graphene nanoplatelets (GnPs) with various functional groups on the edge selectively.^[^ ^46^ ^]^ When further exposed to oxygen and moisture in the air, hydrogen‐GnPs, sulfonic acid‐GnPs, carboxylic acid‐GnPs, or carboxylic acid/sulfonic acid‐GnPs can be generated. They show excellent performance in electrocatalyst and functional device applications.

##### Microwave assisted reaction

Chemical synthesis of highly modified graphite can also be accomplished using microwave‐sparks‐assisted halogenation reactions (Figure 9D).^[^ ^175^ ^]^ In this case, microwave irradiation may cause the halogen to become slightly ionized, which aids the halogenation reaction. The Cl and Br atoms have been covalently functionalized on the graphene sheet with ∼21% and ∼4% atom percentage. The graphene halides (G─X) can be easily exfoliated into monolayer graphene in organic solvents. After high temperature annealing, the chlorine content is reduced, and the structure of chlorinated graphene (G─Cl) can be restored. As a result, after annealing, the conductivity of the G─Cl film increases significantly. The high solubility and low cost of G─Cl allow it to meet the requirements for large‐area and economical electronic devices such as FETs, photovoltaics, and light‐emitting diodes. For instance, the G─Cl itself can be used as a functional component to prepare FETs. G─X could also be an important versatile precursor and intermediate material to fabricate new composite materials.

PFPA can also be used to functionalize graphene through a microwave‐assisted reaction.^[^ ^176^ ^]^ Under microwave radiation, molecular collisions caused by relatively high‐frequency electromagnetic radiation produce active singlet perfluorophenyl nitrene, which can react with C═C bonds of graphite via cycloaddition. Therefore, graphene can react with PFPA when exposed to microwaves. Any amine‐containing molecules can be covalently attached to the PFPA functionalized graphene. For instance, it can conjugate with glycosyl amine to give carbohydrate‐presenting graphene.

## 5 CONCLUSION AND PERSPECTIVES

Graphene has been used in a wide range of applications, since it can be effectively exfoliated, tailored, and modified. Whether in laboratory research or industrial applications, large‐scale, high‐quality, and layer‐controlled graphene with specific functionalities is a worthy goal to pursue.

As two parallel roads for graphene preparation, CVD and mechanical exfoliation provide strong support for the availability of large‐area and high‐quality graphene based on their complementary advantages, holding great prospects for the realization of graphene‐based integrated circuits and the exploration of novel phenomenon, such as superconductivity discovered in magic‐angle graphene superlattices. Much effort should be made to improve the quality and size of graphene. In case of CVD, growth conditions are needed to be optimized to obtain high‐quality single‐crystal graphene on metal substrates. Gentle and clean transfer procedures are required to preserve the excellent transport properties of graphene. New methods are needed to be developed to achieve graphene growth directly on dielectric surfaces. Furthermore, mechanism studies of graphene exfoliation are needed to be explored, which is crucial for obtaining large‐area and high‐quality graphene.

Etching is a key step to fabricate various structures of graphene, endowing graphene with excellent properties. Traditional organic synthesis is also seen as a complementary choice for producing specific pattern of graphene or other materials, but involves complicated condition control and product post‐processing. In contrast, top‐down etching can effectively control the morphology of graphene over large area in combination with EBL, and anisotropic etching provide scientists with an alternative to studying topological and spin properties of edge states. However, it should be noted that an in‐depth understanding of the etching mechanism and the establishment of the theoretical model are required to study the underlying effects of different etching conditions.

Through covalent functionalization, molecular catalysts can be immobilized on the graphene surface to form stable photocatalytic materials. In graphene chemistry, most reactions on graphene originate from the organic reactions of benzene. However, it is not rigorous enough to regard graphene as benzene, so the reaction mechanism on graphene needs to be further studied in the future. It is also necessary to precisely control the modified positions of graphene according to specific requirements, such as opening the bandgap or implementing new mechanisms. Target molecules should be introduced to realize the specific functions of graphene‐based devices.

On the basis of improved graphene preparation, tailoring, and modification methods, large‐area and high‐quality graphene with precisely controlled patterns and specific functional groups should be used to fabricate devices. These three parts will invite in‐depth research on the amazing structures and electronic devices of graphene, such as magic‐angle graphene and graphene‐based single‐molecule junctions, to satisfy the exploration of novel properties and applications of graphene. Through delicate design, the processes of preparation, tailoring, and modification should be further combined to fabricate next‐generation electronic devices.

## CONFLICT OF INTEREST

The authors declare no conflict of interest.
